# Sleep Disturbance, Irritability, and Response to Lurasidone Treatment in Children and Adolescents with Bipolar Depression

**DOI:** 10.2174/1570159X20666220927112625

**Published:** 2023-05-12

**Authors:** Manpreet K. Singh, Cynthia Siu, Michael Tocco, Andrei Pikalov, Antony Loebel

**Affiliations:** 1 Department of Psychiatry and Behavioral Sciences, Stanford University, Stanford, CA, USA;; 2 COS and Associates Ltd., Central, Hong Kong, and Toronto, Ontario, Canada;; 3 Sunovion Pharmaceuticals Inc., 500 Frank W Burr Blvd, Suite 550, Teaneck, NJ, and Marlborough, MA, USA

**Keywords:** Mixed mood, hypomania, depression, lurasidone, bridge symptoms, network analysis

## Abstract

**Background:**

The presence of mixed (subsyndromal hypomanic) symptoms may influence treatment outcomes in pediatric bipolar depression. This post-hoc analysis investigated “bridge” symptoms that have cross-sectional and predictive associations with depressive and manic symptom clusters in youth with bipolar depression.

**Methods:**

The moderating effects of these bridge symptoms on the response to flexibly dosed lurasidone 20-80 mg/d compared to placebo treatment was analyzed in children and adolescents with bipolar I depression in a six-week, placebo-controlled, double-blind study followed by a 2-year, open-label extension study of lurasidone.

**Results:**

Sleep disturbances, assessed by “difficulty with sleep” (Children’s Depression Rating Scale, Revised [CDRS-R] item 4) and “decreased need for sleep” (Young Mania Rating Scale [YMRS] item 4), and “irritability” (CDRS-R item-8, YMRS item 5) were identified as “bridge” symptoms and found to have replicable causal associations with depressive (CDRS-R total) and manic symptom clusters (YMRS total) at baseline and week-6. A greater improvement in overall depression severity at week 6 with lurasidone (*vs*. placebo) treatment was observed in the presence (*vs*. absence) of decreased need for sleep at study baseline, mediated in part by significant reductions from study baseline in decreased need for sleep and manic symptom severity. The absence of sleep disturbance and irritability in patients at open-label extension study baseline was associated with higher rates of sustained recovery (symptomatic and functional remission) over 6 months compared to patients with those symptoms at baseline (68% *vs*. 50%, Number Needed to Treat=6).

**Conclusion:**

Our findings suggest that sleep disturbance and irritability are cardinal symptoms that “bridge” between depressive and manic symptom clusters and influence treatment outcomes in youth with bipolar depression.

## 1 INTRODUCTION

The co-occurrence of depressive and manic symptoms in the same mood episode presents significant challenges for the diagnosis and treatment of people with bipolar disorder. The presence of subthreshold hypomanic symptoms in pediatric bipolar depression may be associated with significant impairment in functioning, and can complicate the illness course and treatment outcome [1, 2]. Recognizing the importance of mixed states during depressive episodes in characterizing the course of illness and response to treatment in either bipolar or unipolar depression, the Diagnostic and Statistical Manual 5^th^ edition (DSM-5) added “mixed features” specifiers to episodes of major depression, hypomania or mania. The importance of assessing manic symptoms during episodes of major depression is underscored by evidence that depressive mixed states are associated with greater illness severity, increased risk of suicide, and poor response to lithium [3]. Further, subthreshold manic symptoms that accompany a major depressive episode may worsen when taking antidepressants [4-6], complicating treatment selection due to concerns about treatment-emergent activation.

Mechanisms underlying mixed symptom presentations are complex. The clinical presentations of depression with manic or hypomanic symptoms are highly heterogeneous [7, 8]. In the Systematic Treatment Enhancement Program for Bipolar Disorder (STEP-BD) study, 1030 unique symptom profiles were identified among 3703 depressed outpatients, with 501 profiles (48.6%) demonstrated by only one participant [7]. An analysis of mixed symptom profiles identified 114 unique combinations of 2 to 6 Young Mania Rating Scale (YMRS) items [9] in 208 major depressive disorder (MDD) participants with subthreshold mixed manic/hypomanic features [8]. Understanding how symptom profiles influence treatment courses and outcomes in youth with bipolar depression represents an important goal and may be helpful for treatment selection and planning.

A symptom network can be represented by a collection of nodes (symptoms) and edges (lines linking between symptoms) [10]. Edges in the network graph represent partial correlations or connections between symptoms [10]. In patients with mixed mood states, overlapping “non-specific” symptoms that are causally linked in both depression and manic states from a network perspective could “bridge” the symptom clusters of these disorders [8]. The treatment or deactivation of these “bridge” symptoms has the potential to break the links between mood symptom clusters, generating cascading effects that could result in an overall improvement in mixed mood states [8, 10].

Lurasidone is a second-generation antipsychotic agent with a high affinity for D_2_, 5-HT_2A_, and 5-HT_7_ receptors as an antagonist [11]. In a 6-week, placebo-controlled, double-blind clinical trial, flexibly-dosed lurasidone 20-80 mg/d demonstrated antidepressant effects in children and adolescents with bipolar I depression [12]. The number (YMRS item score > 2 on 2 or more items) and severity (YMRS total score > 5) of the mixed (subsyndromal hypomanic) features at study baseline did not moderate the effect size for improvement in depression severity with lurasidone treatment [13]. However, limited understanding exists regarding the relationship between sleep disturbance, irritability, and their individual or combined effects on outcomes of pharmacotherapy in the treatment of children and adolescents with bipolar depression [14].

A recent network analysis involving adults with subthreshold manic symptoms accompanying a major depressive episode showed sleep disturbance that overlapped and bridged between the depressive and manic symptom clusters at study baseline predicted overall treatment outcomes, as assessed by change from baseline in Montgomery-Asberg Depression Rating Scale (MADRS) [15] and YMRS total scores [9] at week-6 study endpoint [8].

The objectives of this post-hoc analysis were to investigate “bridge” symptoms (overlapping symptoms that have cross-sectional and predictive associations with depressive and manic symptom clusters) in children and adolescents with bipolar I depression and to evaluate their effects on the treatment outcomes of lurasidone 20-80 mg/d in a 6-week, placebo-controlled, double-blind study [12] followed by a 2-year, open-label, extension study of lurasidone treatment [16].

## 2 METHODS

### 2.1 Participants

Study participants were 347 children and adolescents with bipolar I depression enrolled in a previously published randomized placebo-controlled trial of flexibly-dosed lurasidone 20-80 mg/d followed by a two-year, open-label extension study in which participants were either continued on lurasidone or switched from placebo to lurasidone 20-80 mg/d, flexibly dosed (Clinicaltrials.gov identifier: NCT02046369, NCT01914393) [12, 16]. Youth, 10-17 years of age, with a DSM-5 diagnosis of bipolar I depression, with or without rapid cycling disease course, and psychotic features, were randomized to 6 weeks of double-blind treatment with once-daily flexible doses of lurasidone 20-80 mg or placebo in the acute treatment study phase. Eligible participants were required to have a YMRS [9] score of < 15, with a YMRS item 1 (elevated mood) score < 2 at screening and baseline. The bipolar I disorder diagnosis was verified by a trained clinician at the time of screening, by completion of the Schedule for Affective Disorders and Schizophrenia for School-age Children [K-SADS-PL] semi-structured clinical interview [17]. A trained clinician confirmed and documented the current depressive episode associated with bipolar I disorder [12].

### 2.2 Clinical Assessment of Subthreshold Manic Symptoms

The outcome measures in this post-hoc analysis were assessed by the Children’s Depression Rating Scale, Revised (CDRS-R) [18], YMRS [9], and the Children’s Global Assessment Scale (CGAS) [19]. The recovery outcome was defined as meeting the criteria for both symptomatic remission (CDRS-R total score ≤ 28) and functional remission (CGAS score ≥ 71) [20]. All symptom assessments were administered by qualified raters with demonstrated inter-rater reliability [12].

### 2.3 Statistical Analysis

We investigated “bridge” symptoms in a network model that were highly associated with depressive (assessed by 17 CDRS-R item scores) and manic (assessed by 11 YMRS item scores) syndromes. In this analysis, “bridge” symptoms were defined based on the following pre-specified criteria [8]: 1) overlapping symptoms that are prevalent and cut across DSM-5 diagnostic criteria for depression and mania, and 2) had *cross-sectional* and *predictive* associations with the overall depressive and manic symptom clusters. The cross-sectional association was evaluated by whether a higher overall severity of depressive and manic symptom cluster was observed in participants who had both of the overlapping (“bridge”) symptoms compared to participants who did not. The predictive association was established when “bridge” symptoms at baseline predicted treatment outcomes of overall depressive symptom cluster and response trajectories of bipolar depression with lurasidone (*vs*. placebo) treatment.

In the network analysis, the graphical Least Absolute Shrinkage and Selection Operator (LASSO) method [21-23] combined with the Extended Bayesian Information Criterion (EBIC) [24] was applied to estimate 378 pairwise partial correlations (edges) for the 28 symptom nodes (17 CDRS-R items and 11 YMRS items) and to select an optimal parsimonious network model through regularization [21-24]. Both model selection and regularization were performed to obtain an optimal, sparse estimate of the partial correlation matrix for the direct pairwise association between two symptoms after controlling for the influence of all other symptoms in the network [21, 22].

We used clinical trial data and the statistical interaction test for “bridge” symptoms by treatment to evaluate the associations of “bridge” symptoms with treatment outcomes in Analysis of Covariance (ANCOVA) and mixed model for repeated measures (MMRM). The residual symptoms of “decreased need for sleep“ (week-6 YMRS item 4 > 0 *vs*. = 0 absent) and “irritability” (week-6 YMRS item 5 > 0 *vs*. = 0 absent) were dichotomized and compared using MMRM for the continuous outcomes and Generalized Estimating Equation method (GEE) for binary remission data. All statistical tests were two-sided. Nominal *p* values were reported without correction for multiple comparisons because of the exploratory, hypothesis-generating nature of this post-hoc analysis.

## 3 RESULTS

Baseline characteristics were similar for the lurasidone and placebo treatment groups (Table **1**).

The two most prevalent manic symptoms at study baseline in this sample of youth with bipolar depression were “decreased need for sleep” at 53.1% (182/343) (YMRS item 4 > 0) and “irritability” (YMRS item 5 > 0) at 84.0% (288/343). These two symptoms were followed in prevalence by: “disruptive-aggressive behavior” (YMRS item 9 > 0) at 38.8% (133/343), “reduced insight” (YMRS item 11 > 0) at 24.8% (85/343), and “appearance of mania” (YMRS item 10 > 0) at 22.7% (78/343). The remaining YMRS symptom items were observed in less than 15% of the study participants.

### 3.1 Evaluating Cross-sectional Associations of “Bridge” Symptoms with Depressive and Manic Symptom Clusters in the Acute Treatment Phase

A total of 166 (48.4%) youth in this study sample reported having both “difficulty with sleep” (CDRS-R item 4 > 2) and “decreased need for sleep” (YMRS item 4 > 0) at study baseline (*P* < 0.001, chi-square test for association = 23.7). The “decreased need for sleep” (YMRS item 4) symptom was strongly correlated with “difficulty with sleep” (CDRS-R item 4) and “irritability” (YMRS item 5) (both *P* < 0.001).

The overlap in sleep disturbance symptoms (CDRS-R item 4 > 2 and YMRS item 4 > 0) was associated with the co-occurrence of depressive and manic symptom clusters. Fig. (**1**) illustrates the subgroup with overlap sleep disturbance symptoms (n = 166) at study baseline showing higher symptom severity in both depressive (mean CDRS-R total = 59.31, SE = 0.62) and manic symptom clusters (mean YMRS total = 6.95, SE = 0.25), while the absence of decreased need for sleep was associated with significantly lower YMRS total (mean 3.55, SE 0.27; 95% CI for presence *vs*. absence of overlapped symptoms 2.66, 4.13) in participants with predominantly depressive symptoms (mean CDRS-R total 60.51, SE 0.79 for participants with CDRS-R item 4 > 2 and YMRS item 4 = 0 at baseline, n = 114).

A total of 318 (92.7%) youth in this study sample had irritability (CDRS-R item 8 > 2). The overlap in irritability symptoms (CDRS-R item-8 > 2 and YMRS item 5 > 0) was associated with the co-occurrence of depressive and manic symptom clusters. The subgroup with overlap irritability symptoms (n = 277) at study baseline had higher symptom severity in both depressive (mean CDRS-R total = 58.85, SE = 0.50) and manic symptom clusters (mean YMRS total = 6.14, SE = 0.19), while the absence of manic symptom of “irritability” (YMRS item 5 = 0) occurred in only 41 youth and had significantly lower YMRS total (mean 1.02, SE 0.36; 95% CI for presence *vs*. absence of overlapped symptoms 4.12, 6.11) in participants with predominantly depressive symptoms (mean CDRS-R total 59.63, SE 1.35; CDRS-R item 8 > 2 and YMRS item 5 = 0, n = 41).

### 3.2 Evaluating Moderating Effects of Sleep Disturbance and Irritability in the Acute Treatment Phase

Compared to placebo, lurasidone treatment significantly improved “decreased need for sleep” (YMRS item 4) (Cohen’s *d =* 0.29, *P =*0.009) and “difficulty with sleep” (CDRS-R item 4) from baseline to week 6 endpoint (Cohen’s *d =* 0.43, *P* < 0.001).

**Fig. (2):**
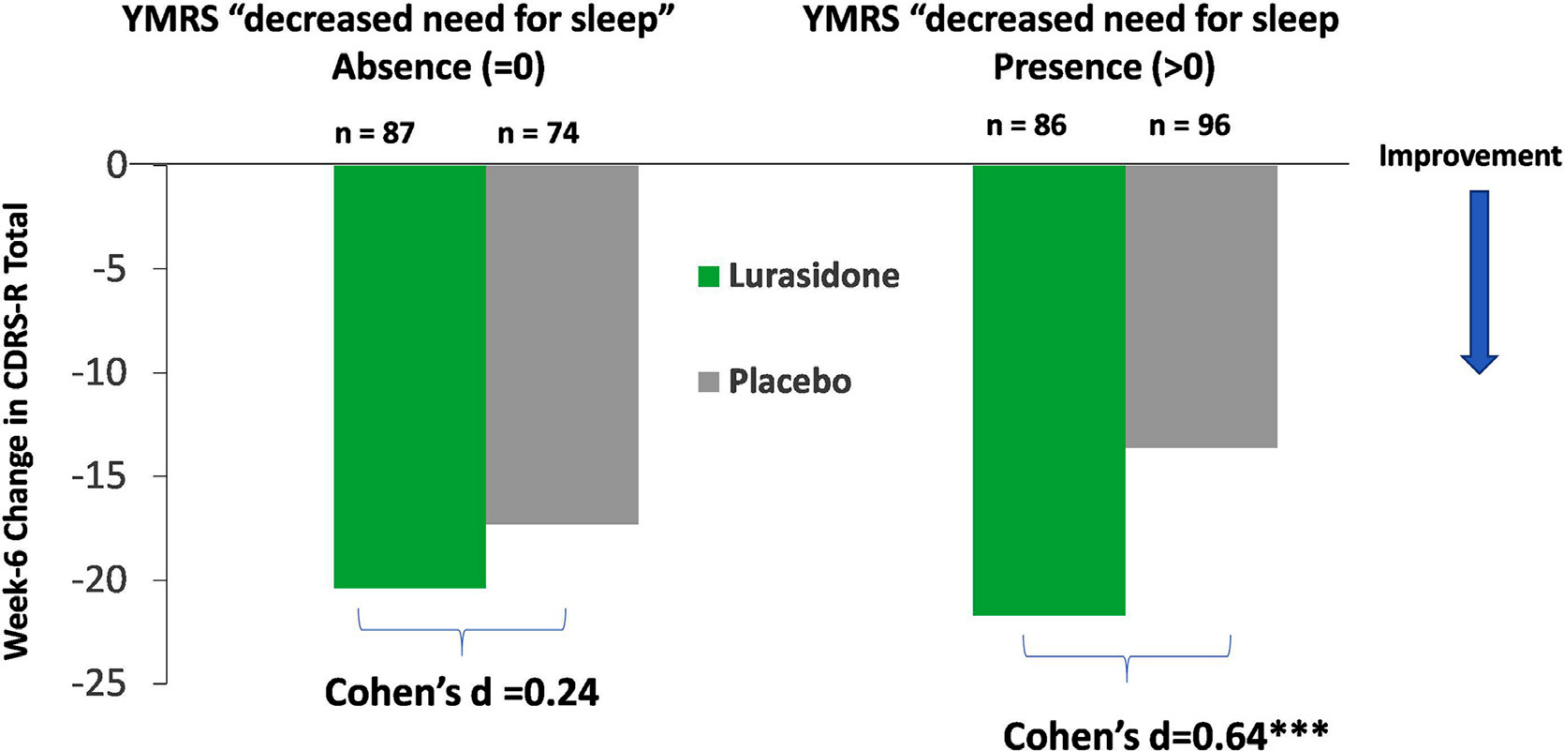
Baseline “decreased need for sleep” (YMRS item 4) and improvement in overall depressive symptom cluster at week-6 endpoint with lurasidone (*vs*. placebo) treatment. **Legend**: Baseline decreased need for sleep moderated change in CDRS-R total score with lurasidone (*vs*. placebo) treatment, *P =* 0.033 for treatment interaction effect with baseline decreased need for sleep. ***P < 0.001 for significant lurasidone (*vs*. placebo) treatment effect on change in CDRS total score observed in participants with “decreased need for sleep” symptom (YMRS item 4 score > 0) at baseline, but non-significant for participants without the “decreased need for sleep” symptom (YMRS item 4 = 0).

The baseline decreased need for sleep predicted a change in CDRS-R total score at the week 6 endpoint with lurasidone (*vs*. placebo) treatment (P = 0.033 for treatment interaction effect with baseline YMRS item 4, F = 4.59, df =1, 330) (Fig. **2**). The presence of “decreased need for sleep” (YMRS item 4 > 0) at study baseline was associated with greater improvement in CDRS-R total score with lurasidone (*vs*. placebo) treatment (Cohen’s *d =* 0.64) from baseline to week 6 compared to participants without this symptom (YMRS item 4 = 0, absent, Cohen’s *d =* 0.24) (Fig. **2**).

Change in YMRS total (*P* < 0.001, *F* = 47.56, *df* = 1, 329) or “decreased need for sleep” (YMRS item 4, *P* < 0.001, *F* = 12.51, *df* = 1, 329) mediated changes in CDRS-R total score with lurasidone treatment.

The presence of “decreased need for sleep” (YMRS item 4 > 0) at study baseline was associated with greater improvement in YMRS total score (Cohen’s *d =* 0.30) with lurasidone (*vs*. placebo) treatment than in the absence of “decreased need for sleep” (YMRS item 4 = 0, Cohen’s *d =* 0.16). The presence of “difficulty with sleep” (CDRS-R item 4 > 2) at study baseline was also associated with greater improvement in YMRS total score (Cohen’s *d =* 0.44) with lurasidone (*vs*. placebo) treatment compared to the participants without this symptom (CDRS-R item 4 < 2, Cohen’s *d =* 0.01) (*P =* 0.050 for the treatment interaction effect with baseline CDRS-R item 4, *F* = 3.86, *df* = 1, 330).

The presence of “decreased need for sleep” (YMRS item 4 > 0) at study baseline was associated with greater improvement in CGAS (Cohen’s *d =* 0.63) from the baseline to week 6 with lurasidone (*vs*. placebo) treatment compared to the participants without this symptom (YMRS item 4 = 0, absent, Cohen’s *d =* 0.16) (*P =* 0.034 for the treatment interaction effect with baseline YMRS item 4, *F* = 4.53, *df* = 1, 325).

**Fig. (3):**
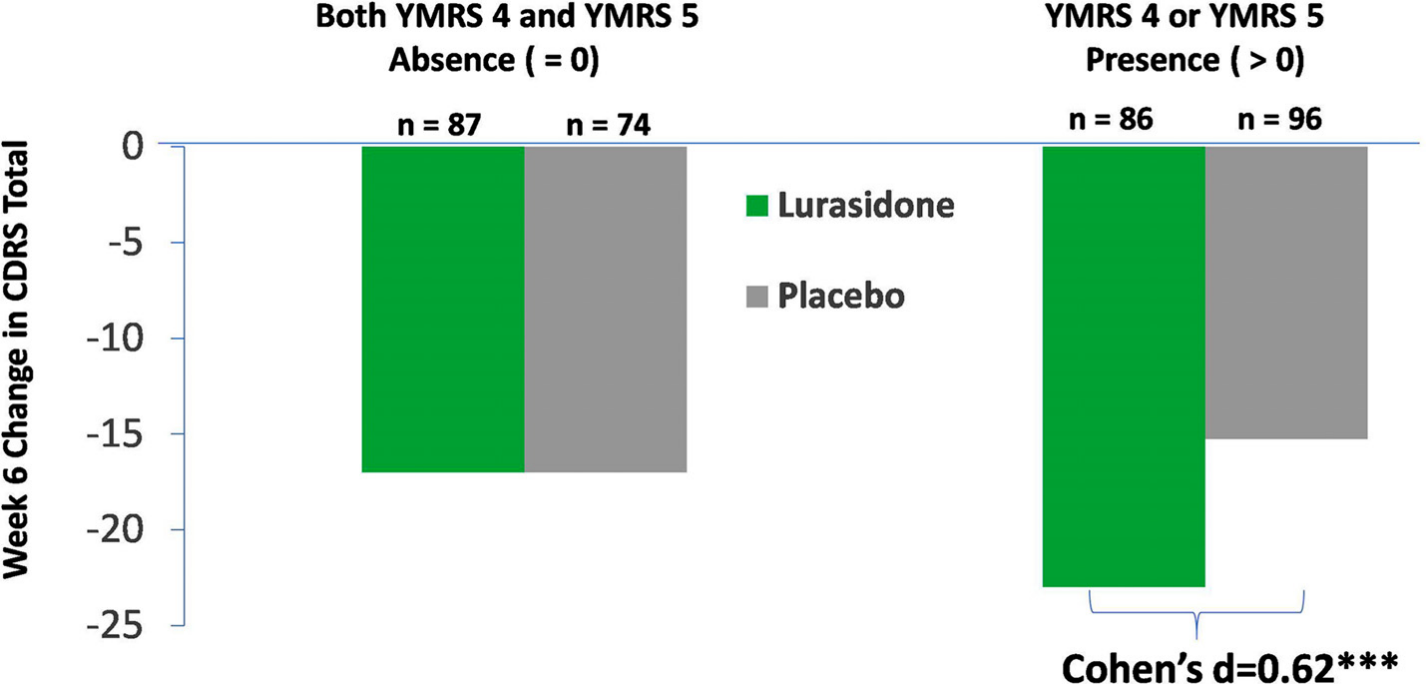
Decreased need for sleep (YMRS item 4) and “irritability” (YMRS item 5) at study baseline and improvement in overall depressive symptom cluster at week-6 endpoint with lurasidone (*vs*. placebo) treatment. **Legend**: Baseline decreased need for sleep combined with “irritability” moderated lurasidone (*vs*. placebo) treatment effect on change in CDRS-R total score: *P* = 0.013 for treatment interaction effect with baseline “decreased need for sleep” (YMRS item 4) and “irritability” (YMRS item 5), both absent or otherwise at study baseline. ***P < 0.001 for significant lurasidone (*vs*. placebo) treatment effect on change in CDRS total score observed in participants with “decreased need for sleep” (YMRS item 4 > 0) or “irritability” (YMRS item 5 > 0) at study baseline, but non-significant for participants without both symptoms (YMRS item 4 = 0 and YMRS item 5 = 0).

The presence of “decreased need for sleep” (YMRS item 4) and/or “irritability” (YMRS item 5) at study baseline moderated the effect of lurasidone (*vs*. placebo) treatment on change in CDRS-R total score at week 6 (Cohen’s *d =*0.62) compared to participants without these two manic symptoms at baseline (*P*= 0.013, *F*= 6.20, *df* = 1, 330, for treatment interaction effect with baseline YMRS items 4 and 5, both absent or otherwise at baseline) (Fig. **3**).

The symptom network structure at the week 6 study endpoint showed a separation of the CDRS-R and YMRS items forming two distinct symptom clusters, while the direct links between “decreased need for sleep” (YMRS item 4) and “difficulty with sleep” (CDRS-R item 4) and between “irritability” YMRS item 5 and CDRS-R item 8 remained (see Graphical Abstract).

### 3.3 Evaluating Moderating Effects of Sleep Disturbance and Irritability in the Two-Year Open-Label Extension Study

**Fig. (4):**
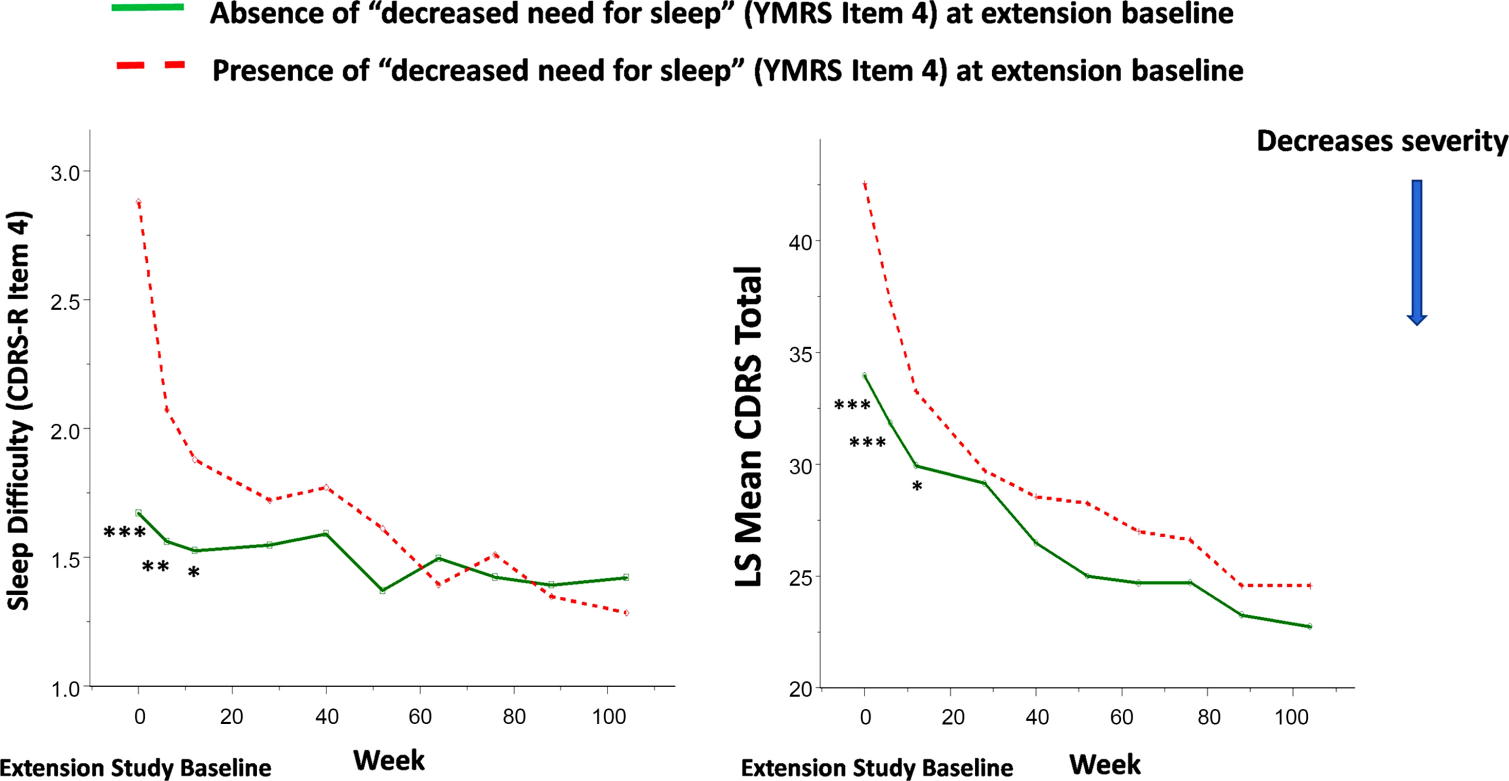
“Decreased need for sleep” (YMRS item 4) at extension baseline and improvement in sleep difficulty and overall depressive symptom severity in youth participants treated with lurasidone in both acute 6-week and 2-year open-label extension studies. Legend: *P<0.05, **P<0.01, ***P<0.001 for present *vs*. absent of “decreased need for sleep” symptom (YMRS Item 4) at extension study baseline.

**Fig. (5):**
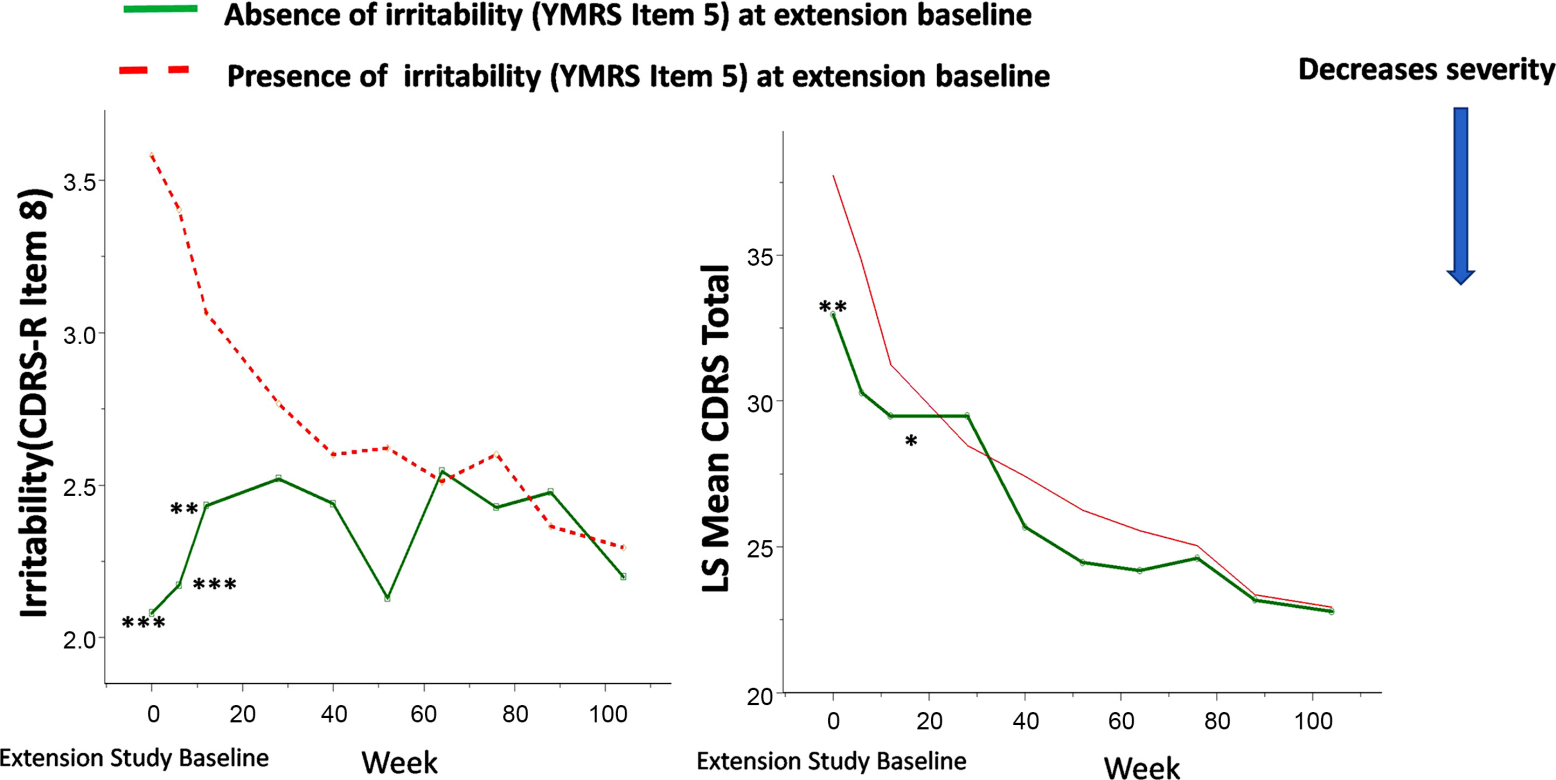
“Irritability” (YMRS item 5) at extension baseline and improvement in overall depressive symptom severity in youth participants treated with lurasidone in both acute 6-week and 2-year open-label extension studies. Legend: *P<0.05, **P<0.01, ***P<0.001 for present *vs*. absent of “irritability” (YMRS item 5) symptom at extension study baseline.

Lurasidone showed continued improvement in decreased need for sleep and irritability, as well as the overall CDRS-R and CGAS scores in the open-label extension study (Figs. **4**, **5**). An improvement in decreased need for sleep and irritability was associated with changes in CDRS-R and CGAS scores over a two-year open-label maintenance treatment with lurasidone.

**Fig. (6):**
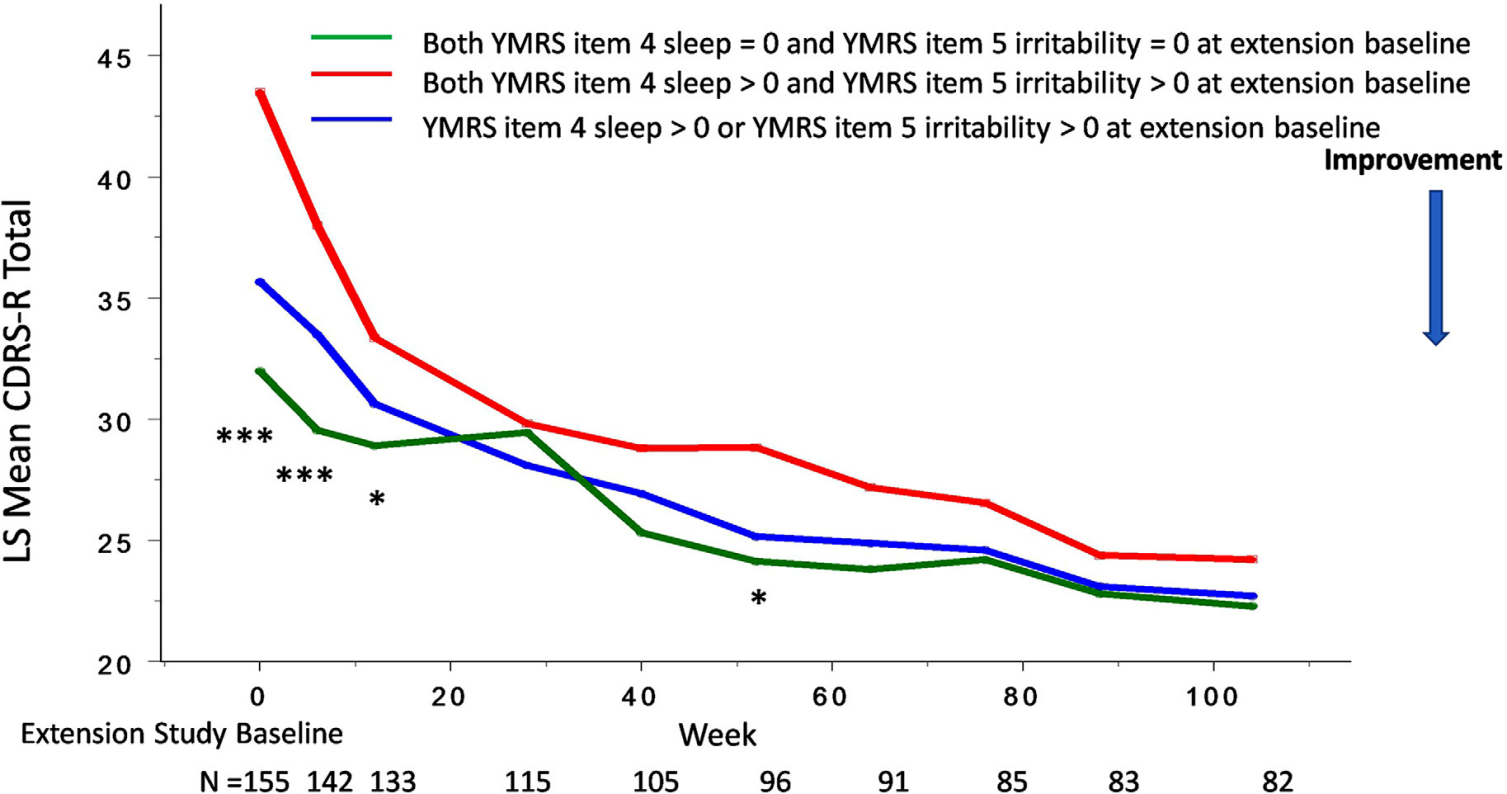
“Decreased need for sleep” (YMRS item 4) and “irritability” (YMRS item 5) at extension baseline and improvement in overall depressive symptom severity in youth participants treated with lurasidone in both acute 6-week and 2-year open-label extension studies. Legend: *P<0.05, **P<0.01, ***P<0.001 for both “decreased need for sleep” (YMRS item 4) and “irritability” (YMRS item 5), both absent *vs*. both present at extension study baseline.

**Fig. (7):**
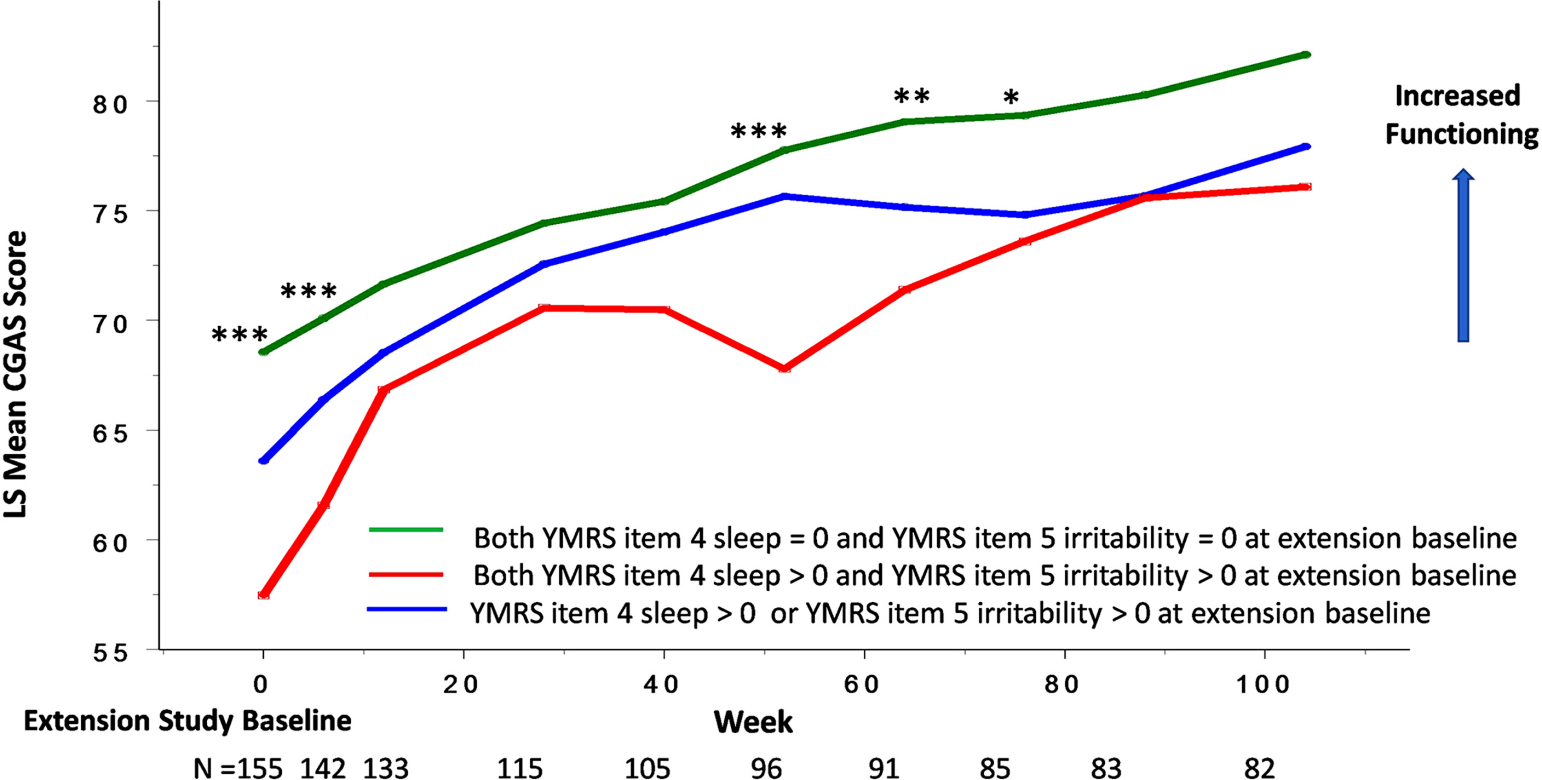
“Decreased need for sleep” (YMRS item 4) and “irritability” (YMRS item 5) at extension baseline and improvement in global functioning in youth participants treated with lurasidone in both acute 6-week and 2-year open-label extension studies. Legend: *P<0.05, **P<0.01, ***P<0.001 for both “decreased need for sleep” (YMRS item 4) and “irritability” (YMRS item 5), both absent *vs*. both present at extension study baseline.

The presence of symptoms involving “decreased need for sleep” (YMRS item 4) and “irritability” (YMRS item 5) at extension baseline predicted trajectories of antidepressant (as assessed by CDRS-R score) and functioning (as assessed by CGAS score) responses to lurasidone treatment during the two-year, open-label extension study (Figs. **6**, **7**).

For the participants who had no symptoms of sleep and irritability (YMRS item 4 =0 and YMRS item 5 =0) at extension study baseline, lurasidone was associated with a long-term maintenance effect on the CDRS-R and CGAS scores observed at baseline of the open-label extension study (Figs. **6**, **7**).

**Fig. (8):**
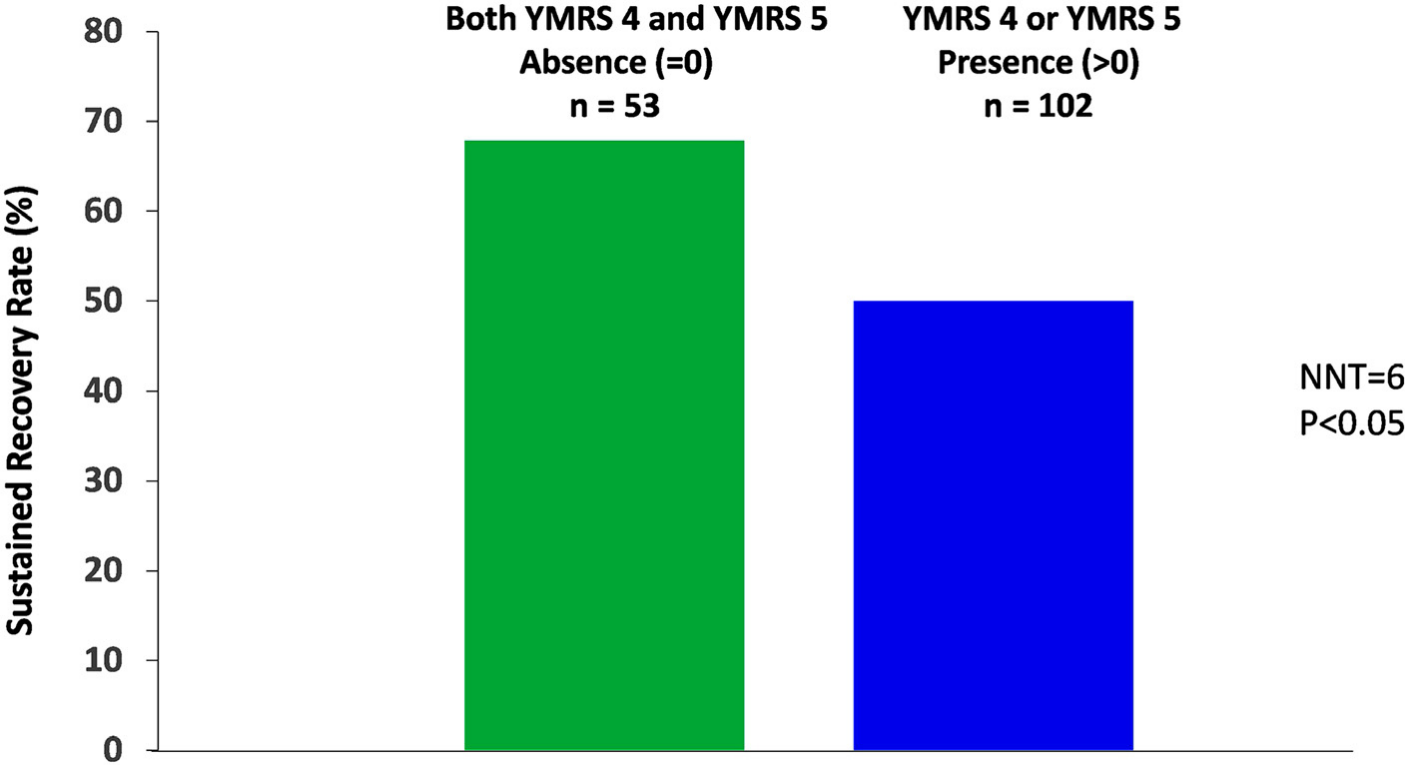
“Decreased need for sleep” (YMRS item 4) and “irritability” (YMRS item 5) at the extension baseline and the rate of sustained recovery in youth participants treated with lurasidone in both acute 6-week and 2-year open-label extension studies. Legend: Sustained recovery was defined as achieving both symptomatic remission (CDRS-R score ≤ 28) and functional remission (CGAS score ≥ 71) criteria for 6 months or longer (2 consecutive visits).

The absence of both “decreased need for sleep” (YMRS item 4) and “irritability” (YMRS item 5) (*vs*. otherwise at extension study baseline) was associated with an increased rate of sustained recovery as assessed by achieving both symptomatic remissions (CDRS-R score ≤ 28) and functional remission (CGAS score ≥ 71) criteria for 6 months or longer (2 consecutive visits) (68% *vs*. 50%, NNT=6) (Fig. **8**).

## 4 DISCUSSION

We investigated “bridge” symptoms (overlapping symptoms that have cross-sectional and predictive associations with depressive and manic symptom clusters) using a network approach in children and adolescents with bipolar I depression based on a placebo-controlled, double-blind study of flexibly-dosed lurasidone 20-80 mg/d followed by a 2-year, open-label extension study of lurasidone treatment [12, 16]. In this post-hoc analysis, sleep disturbance (assessed by CDRS-R item 4 “difficulty with sleep” and YMRS item 4 “decreased need for sleep”) and irritability (assessed by CDRS-R item 8 and YMRS item 5) were identified as “bridge” symptoms based on pre-specified criteria that supported links between depressive and manic symptom clusters [8].

Decreased need for sleep and irritability (“bridge”) symptoms are typically reported as common and disruptive in pediatric bipolar depression [14, 25-28]. Sleep disturbance and irritability which are part of DSM-5 diagnostic criteria for depression and mania/hypomania, from a network perspective, form robust and replicable links (edges) between both the depressive symptom cluster (assessed by CDRS-R total score) and the manic symptom cluster (assessed by YMRS total score) [8, 10, 27]. Targeting these “bridge” symptoms may reduce interactions between depressive and manic symptoms, leading to an improvement in the severity of mixed features, (Graphical Abstract) [8, 10, 14, 25-28]. Further, the dynamic network model of interacting symptoms suggests that improvement in (*i.e*. deactivation of) “bridge” symptoms (decreased need for sleep and irritability) has cascading effects resulting in an overall improvement in the depressive and manic symptom clusters in patients with bipolar depression [7, 8, 10, 27]. This implies that these “bridge” symptoms might be a useful therapeutic target for bipolar depression syndrome.

Youth who had difficulty with sleep and decreased need for sleep had higher YMRS and CDRS-R total scores at baseline and at week-6 endpoints. The subgroup with irritability symptoms (both CDRS-R item 8 > 2 and YMRS item 5 > 0) had higher severity of depressive and manic symptoms at baseline and week-6 endpoints. These findings indicated that sleep disturbance and irritability (“bridge symptoms”) had robust and replicable associations with depressive and manic symptom clusters (Graphical Abstract).

The predictive associations for the effects of “bridge” symptoms on the depressive and manic symptom clusters were demonstrated by a significant moderating effect of decreased need for sleep or decreased need for sleep combined with irritability (absent *vs*. present at study baseline) on improvement in CDRS-R and YMRS total score in both acute 6-week as well as long-term treatment with lurasidone. Specifically, the presence (*vs*. absence) of decreased need for sleep or decreased need for sleep combined with irritability symptoms at acute study baseline predicted a larger lurasidone effect size for improvement in overall depressive symptoms at week 6. A reduction in decreased need for sleep and irritability with lurasidone treatment was found to mediate improvement in depressive and manic symptoms at week 6.

For participants who had no symptoms of decreased need for sleep and irritability at extension study baseline, low CDRS-R and high CGAS scores were maintained during lurasidone treatment over a 2-year follow-up period. Among youth with decreased need for sleep and/or irritability at extension study baseline, continued improvement in these symptoms, as well as overall CDRS-R and CGAS scores were observed over the 2-year, open-label study period.

In youth treated with lurasidone during the acute and long-term treatment phases, resolution of “decreased need for sleep” (YMRS item 4 = 0 absent) and “irritability” (YMRS item 5 = 0 absent) symptoms at the week 6 endpoint predicted higher remission rates (CDRS-R total score ≤ 28).

Significantly more participants without symptoms of decreased need for sleep and irritability at the end of the 6-week acute treatment period achieved sustained recovery criteria [20] after two years of lurasidone treatment compared to participants with these symptoms (68% *vs*. 50%, respectively).

Patients with persistent sleep and irritability symptoms at the extension study baseline showed lower rates of remission and functional recovery in the long-term extension study period. Further prospective studies should examine whether treatments to specifically ameliorate sleep disturbance and irritability symptoms could improve the speed and magnitude of overall symptom improvement in youth with bipolar depression.

Collectively, these findings indicate that sleep disturbance and irritability (“bridge”) symptoms at study baseline were causally linked with changes in depressive and manic symptoms at study endpoint. Our findings are consistent with the existing body of evidence that sleep disturbance plays a central role in bipolar disorders and may be functioning as a causal factor in the progression of the disorder [27-32].

Our work [8, 33, 34] and others [35-37] suggest the potential for antioxidant and stress responsivity effects associated with lurasidone treatment. Models of cellular recovery after chronic stress may link sleep regulation to depression severity and recovery. Sleep disturbances may be associated with a high potential for an antioxidant imbalance, in part due to hormonal response to stress, to the activation of the hypothalamic-pituitary-adrenal (HPA) axis, or due to the immune-inflammatory system as it intersects with cognitive, emotional, and behavioral dysfunctions [38-47]. In the context of antioxidant defense responses to sleep loss and sleep recovery [43-45], it is hypothesized that the duration of sleep when adversely affected by stress may be controlled by a homeostatic drive regulated by a group of vitagenes that are involved in preserving cellular homeostasis during stress [38-42, 47]. Activation of these protective redox-dependent vitagene networks (LXA4) block generations of pro-inflammatory cytokines and reactive oxygen species (ROS) [47], thus promoting an antidepressant response and related functional recovery. These findings provide some insights into the biological processes underlying sleep, preconditioning signals and hormesis, the antioxidant defense system, its interaction with the immune/inflammatory responses, and diverse forms of stress that are common to the disease pathophysiology and recovery processes, leading to the resolution of sleep disturbances, mood symptoms, and functional deficits [38-47].

Sleep disturbance often manifests itself as decreased need for sleep in participants with manic or hypomanic symptoms. Under the sleep-wake homeostasis process, increased periods of wakefulness result in an increased need for sleep. Thus, the co-occurrence of decreased need for sleep and reduced sleep may indicate a mismatched sleep-wake cycle in bipolar disorder [30, 31]. In the absence of “decreased need for sleep” (YMRS item 4) symptom, “difficulty with sleep” (CDRS-R item 4) may assess simple insomnia and seems to be associated primarily with a depressive symptom cluster. In contrast, reduced sleep symptoms in bipolar depression youth with manic or hypomanic features may be a by-product of influences of hyperactivity and irritability that characterize the manic state in youth, and who may commonly present with co-occurring attention deficit with hyperactivity [2, 31]. Sleep disturbance as a “bridge” symptom may therefore be a useful marker of mixed states [8, 14, 27]. These models are consistent with prior studies examining the role of sleep disturbance in adult participants with bipolar disorders [27, 30, 31, 48], suggesting sleep deprivation as a contributing factor to mania. Based on our findings, approximately 50% of youth participants experienced difficulty with sleep and decreased need for sleep during a bipolar depressive episode. Those same participants also had higher YMRS total scores compared to participants with only the difficulty with sleep symptom but not the decreased need for sleep symptoms. Therefore, interventions targeting “bridge” symptoms, especially decreased need for sleep, may help reduce mixed (subthreshold hypomanic) features during a bipolar depressive episode.

Results from the current study in children and adolescents with bipolar I depression are consistent with a prior study examining “bridge” symptoms that connected manic (11 YMRS items) and depressive (10 MADRS items) symptom clusters in adult participants with MDD presenting with two or three manic symptoms (DSM-5 mixed features specifier) [8]. The network symptom structure of MDD with mixed features suggested that sleep disturbance assessed by “reduced sleep” (MADRS item 4) and “decreased need for sleep” (YMRS item 4) overlapped and strongly connected to each other. These symptoms also had predictive associations with the depressive and manic symptom clusters. In the adult MDD study, the presence of “rapid/pressured speech” (YMRS item 6), which directly linked reduced sleep to the manic symptom cluster at study baseline, predicted greater improvement in overall depressive symptoms mediated by manic symptom reduction. There were direct causal links (LASSO regularized partial correlations) from decreased need for sleep to “elevated mood” (YMRS item 1) and “increased motor energy” (YMRS item 2) and depressive symptom cluster, such that the absence of these core manic symptoms at baseline predicted greater improvement in depressive symptom cluster with lurasidone (*vs*. placebo) treatment than their presence in participants with predominantly depressive symptoms.

Both sleep disturbance and irritability were robustly linked to depressive and manic symptom clusters before and after randomized treatment. We show that replicable network association structures based on “bridge” symptoms may be useful for making causal inferences about their effects on short and long-term treatment outcomes. These findings are consistent with the “bridge” symptoms that linked decreased need for sleep and reduced sleep in adult participants with MDD presenting with mixed (subthreshold hypomanic) features [8].

### 4.1 Limitations

It is well known that the symptom network structure based on cross-sectional correlations between symptoms (edges) can be sensitive to small variations in participant samples due to the interrelated nature of psychopathology symptoms [8, 29]. Thus, we focused on the replicable association structures of “bridge” symptoms that often overlap and belong in the diagnostic criteria of depression, mania, and hypomania.

Many YMRS items were rated 0 (absent) for some youth in this study. Relatively low base rates may have affected the adequate evaluation of these subthreshold (manic/hypomanic) features in the symptom network model. We investigated the combined effects of sleep disturbance and irritability in the acute study phase because over 90% of participants in this study had irritability at baseline. Consequently, it was challenging to separate the effects of irritability from sleep disturbance on treatment outcomes at week-6 in this dataset.

Apart from sleep disturbance, other manic symptoms might also affect treatment responses in bipolar I depression in youth. The criterion based on the number (YMRS item score > 2 on 2 or more items) and severity (YMRS total score > 5) of the mixed (subsyndromal hypomanic) features did not predict treatment outcomes in this study sample, as assessed primarily by change in overall depression severity [13]. Consistent with this, the number and severity of mixed features were not found to be modifiers of treatment responses in adults with mixed manic/hypomanic symptoms co-occurring during MDD or bipolar depression [8, 13, 49]. Future studies should further explore the impact of other manic/hypomanic symptoms in the context of a depressive episode. Finally, in this post-hoc analysis, we dichotomized the decreased need for sleep and irritability symptoms into the categories of present *vs*. absent subgroups when evaluating their moderating effects as “bridge” symptoms on treatment outcomes. However, we observed similar results in sensitivity analyses using continuous dimensions of these symptoms.

## CONCLUSION

The treatment of bipolar depressive symptoms in children and adolescents is challenging given the complexities of the diagnosis and its associated phenomenology with a need to improve clinical outcomes. This post-hoc analysis of a placebo-controlled, six-week, double-blind trial of lurasidone (*vs*. placebo) followed by a 2-year, open-label extension study in children and adolescents with bipolar depression demonstrated that sleep disturbance and irritability constituted cardinal symptoms that bridge between depressive and manic symptom clusters. In our network model, improvement (deactivation) in decreased need for sleep and irritability symptoms resulted in a decreased overall depressive and manic symptom cluster severity. These findings suggest that treatment of sleep disturbance and irritability symptoms can influence treatment outcomes in children and adolescents with bipolar depression.

## Figures and Tables

**Fig. (1):**
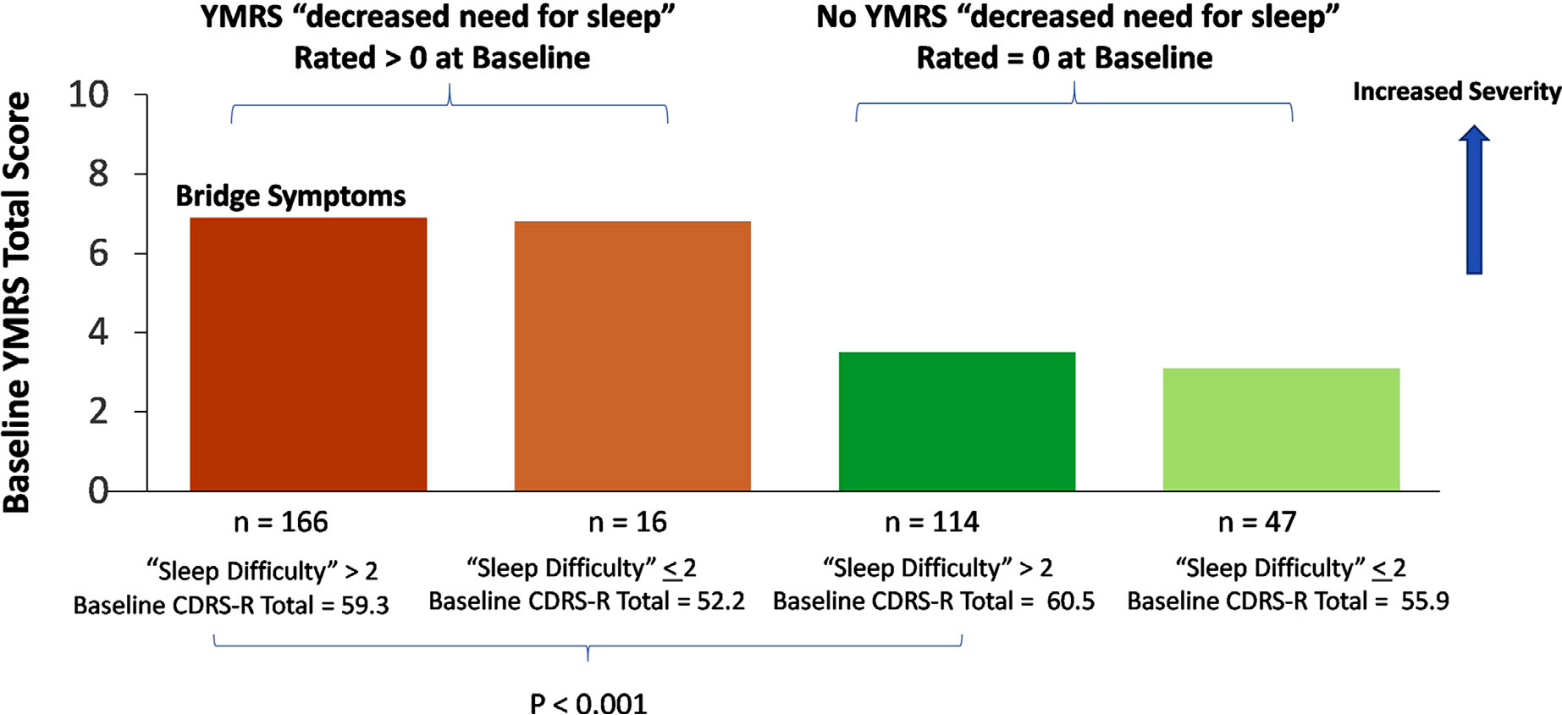
Associations of sleep disturbance symptoms (“bridge symptoms”) with depressive and manic symptom clusters in children and adolescents with bipolar I depression.

**Table 1:** The demographic and clinical characteristics of the intent-to-treat study sample at baseline.

**Characteristic**	**Lurasidone 20-80 mg/d (N = 173)**	**Placebo** **(N = 170)**
Male, %	50.9	51.2
Age, years, mean ± SD	14.2 ± 2.2	14.3 ± 2.0
Race % White Black/African American Other	77.5 8.7 13.9	73.5 10.6 15.9
ADHD diagnosis, %	23.7	21.8
ADHD treated with Stimulants, %	10.4	12.4
Baseline Scores Double-blind/Open-label, mean ± SD		
CDRS-R Total Score	59.2 ± 8.2 / 36.6 ± 12.5	58.6 ± 8.3 / 41.9 ± 13.8
CGI-BP-Depression Score	4.6 ± 0.6 / 3.0 ± 1.1	4.5 ± 0.6 / 3.4 ± 1.1
CGAS Score	48.8 ± 8.7 / 63.1 ± 12.4	49.5 ± 7.0 / 58.9 ± 12.5
Young Mania Rating Scale	5.5 ± 3.8 / 3.3 ± 3.3	5.1 ± 3.2 / 3.7 ± 4.3

## Data Availability

Not applicable.

## LIST OF ABBREVIATIONS

CDRS-R: Children’s Depression Rating Scale, Revised
CGAS: Children’s Global Assessment Scale
EBIC: Extended Bayesian Information Criterion
LASSO: Least Absolute Shrinkage and Selection Operator
MDD: Major Depressive Disorder
STEP-BD: Systematic Treatment Enhancement Program for Bipolar Disorder
YMRS: Young Mania Rating Scale
